# *Leishmania amazonensis* infection impairs dendritic cell migration from the inflammatory site to the draining lymph node

**DOI:** 10.1186/1471-2334-14-450

**Published:** 2014-08-20

**Authors:** Micely DR Hermida, Priscila G Doria, Angela MP Taguchi, José O Mengel, Washington LC dos-Santos

**Affiliations:** Centro de Pesquisas Gonçalo Moniz, Oswaldo Cruz Foundation, Fiocruz, LPBI, Salvador, Brazil; Instituto Oswaldo Cruz, Oswaldo Cruz Foundation, Fiocruz, Rio de Janeiro Brazil; Faculty of Medicine of Petrópolis, FMP-FASE, Petrópolis, Brazil

**Keywords:** *Leishmania*, Leukocyte migration, Dendritic cell, Lymph node, Macrophage

## Abstract

**Background:**

*In vitro* studies show that *Leishmania* infection decreases the adhesion of inflammatory phagocytes to connective tissue by a mechanism dependent on the modulation of integrin function. However, we know little about the influence of this reduction in leukocyte adhesion on parasite dissemination from the infection site.

**Methods:**

In this work, we used a model of chronic peritonitis induced by thioglycollate to study the effect of *L. amazonensis* infection on the ability of inflammatory phagocyte populations to migrate from an inflammatory site to the draining lymph node. Uninfected or *Leishmania*-infected thioglycollate-elicited peritoneal exudate cells were transferred from C57BL/6 to BALB/c mice or from Ly5.1^+^ to Ly5.1^-^ mice. The transferred cells were injected into the peritoneal cavity and tracked to the draining lymph node.

**Results:**

Migrating cells corresponded to approximately 1% of the injected leukocytes. The proportion of migrating CD11b^+^CD11c^+^ (myeloid dendritic cell) was lower after incubation with *Leishmania* (1.3 to 2.6 times lower in the experiments using C57BL/6 to BALB/c animals and 2.7 to 3.4 times lower in the experiments using Ly5.1^+^ to Ly5.1^-^ animals) than after leukocyte incubation with medium alone (P < 0.01). There was no consistent decrease in the migration of CD11b^+^F4/80^+^ (macrophage) or SSC^hi^ GR-1^+^ (neutrophil) populations.

**Conclusions:**

Coincubation with *Leishmania* changes the migratory pattern of dendritic cells *in vivo*. Such changes in dendritic cell migration may be associated with immunological events that maintain inflammation at the sites of infection.

**Electronic supplementary material:**

The online version of this article (doi:10.1186/1471-2334-14-450) contains supplementary material, which is available to authorized users.

## Background

Leishmaniasis is initiated by the inoculation of *Leishmania* promastigotes into the skin of a susceptible host during the blood meal of an infected sand fly [1]. Thereafter, the parasite may remain at the inoculation site or disseminate in the host tissues. Permanence at the inflammation site and dissemination of the parasite may result in a wide spectrum of clinical manifestations that range from self-healing skin ulcers to disfiguring mucosal lesions and fatal visceral leishmaniasis [1, 2]. At the *Leishmania* inoculation site, various phagocytes have the potential to phagocytize and transport live amastigotes or killed parasites, which may also act as antigens, to the draining lymph node. These cells include dermal resident dendritic cells (DC), epidermal Langerhans cells, neutrophils and monocytes, which are released from the blood when the dermal capillary is disrupted and a reputed blood lake is formed [3–6]. Soon after, neutrophils and macrophages may still be recruited in response to local inflammatory stimuli that persist for 2–3 days [6]. Hence, a variety of mononuclear phagocytes have been shown to bear live amastigotes or molecules of dead parasites, both at the infection site and in the draining lymph node [4, 7]. The ability of Langerhans cells or monocyte-derived DC to transport amastigotes from the skin to the lymph node has been demonstrated in various studies [3, 7]. In progressive forms of cutaneous leishmaniasis, parasites and parasite molecules are continuously observed in the interior of the mononuclear phagocytes in the skin lesion and in the marginal sinus of the draining lymph nodes, suggesting that these phagocytes continuously transport parasite and parasite molecules [4]. At the inoculation site, amastigotes are maintained in the interior of the mononuclear phagocytes, and the immunoreactions elicited by these cells are associated with different types of nodular lesions or ulcers. We still know very little about the mechanisms controlling the ability of these cells containing live parasite or parasite fragments to remain in the infection site or to migrate to the draining lymph node. Evidence suggests that a number of phagocyte functions potentially associated with cell migration, such as intracellular PKC-dependent signaling pathways, cell adhesion and migration toward chemokine stimuli, are modulated during *Leishmania* infection [8–10]. In a previous work, we showed that coincubation with *Leishmania* impairs the adhesion of different mononuclear phagocytes to inflamed connective tissues and to connective matrix components *in vitro* and that this decrease in cell adherence correlates with the proportion of infected phagocytes and the parasite burden in the cell [11, 12].

Leukocyte exit from the inflammatory site towards the lymph node involves a sequence of steps, starting with the response of these cells to chemokines released by lymphatic endothelial cells [13–16]. In response to this stimulus, the leukocyte sequentially modulates the engagement of integrin interaction with connective tissue components in a directional way to reach the periphery of the lymphatic capillary [17, 18]. Both adhesion and detachment are coordinated necessary steps in cell migration. Hence, the loss of adherence by phagocytes with a high *Leishmania* burden may impair the exit of these cells from the inflammatory site. The capability of *Leishmania*-infected cells to migrate or to remain in the infection site may be important for defining the characteristics and lesion distribution in leishmaniasis. For example, most of cutaneous leishmaniasi ulcer develops at the infection site, sometimes months after an initial papule. In other instances, after cure of cutaneous lesion metastatic mucocutaneos ulcers emerges.

In this study, we used the murine model of phagocyte migration *in vivo* described by Bellingan and collaborators [19] to examine whether the decrease in adhesion of phagocytes co-incubated with *Leishmania* to the connective tissue observed *in vitro* corresponded to changes in the migratory pattern of these cells *in vivo*. The model described by Bellingan and colleagues is designed for studying macrophage exit through the lymphatic system during the resolution of a thioglycollate-induced peritonitis and has characteristics that make it suitable for use in our study: (1) the thioglycollate-induced peritoneal exudate contains a variety of phagocytes, including neutrophils, macrophages and dendritic cell populations [11, 20, 21]; (2) these cells are present in large numbers, thus allowing easy collection, *in vitro* manipulation and reinjection; (3) the adherence of the PEC to the connective tissue is also decreased after cultivation with *Leishmania* [11, 12]; and (4) the emigration of mononuclear phagocytes from the peritoneal cavity to the lymph nodes observed in this *in vivo* migration system is dependent on VLA-4 [22], a molecule that we have shown to have altered function in *Leishmania*-infected phagocytes [8].

Bellingan and collaborators showed that the phagocyte exit from the peritoneal cavity to the parathymic lymph node is maximal in the 4^th^ day of thioglycollate peritonitis [19]. Hence, we chose this time point to study the effect of *Leishmania* infection on the migration of different populations of phagocytes to the draining lymph node. In this series of experiments we used *Leishmania amazonensis*. This species is endemic in parts of Brazil, usually associated with cutaneous lesions. However, some *L. amazonensis* strains have also been isolated from patients with visceral leishmaniasis [23]. Infections by this First, we performed a partial characterization of the phagocytes present in the peritoneal exudate and examined the capability of these different cell populations for phagocytizing *Leishmania*. Subsequently, we examined the change in the migration rate of cells incubated with *Leishmania* in conditions favoring the maximal decrease (70%) in peritoneal exudate cell adhesion to connective matrix components observed in our studies *in vitro* [11, 12]. The result of this study may contribute to the knowledge of the factors associated with the permanence of infected cells at the inoculation site or their dissemination to other host tissues.

## Methods

### Animals

Six- to eight-week-old BALB/c, C57BL/6 and C57BL/6 SJL mice (antigenic specificity Ly5.1) of both genders were obtained from the colony of the Gonçalo Moniz Research Center-FIOCRUZ (Salvador, Brazil). The animals were maintained under the controlled environmental conditions of humidity, temperature and a light–dark cycle, with commercial balanced mouse chow and water provided *ad libitum*. The animals were subjected to thioglycollate peritonitis and adoptive cell transfer via intraperitoneal injection (see below). At the end of the experiments, the animals were killed by lethal CO_2_ anesthesia, and the peritoneal exudate cells and parathymic lymph nodes were removed as described below. All experiments involving animals were conducted in accordance with Brazilian Federal Law on Animal Experimentation (Law 11794) [24] (http://www.planalto.gov.br/ccivil_03/_ato2007-2010/2008/lei/11794.htm). This study was approved by the Committee of Ethics in the Use of Animals of the CPqGM-FIOCRUZ (Ceua, licence N. 018/2009).

### Phagocyte infections with *Leishmania*

*Leishmania amazonensis* (Leila strain, MHOM/BR88/BA-125) were grown in Schneider’s insect medium (Sigma) containing 10% FBS at 24°C. Stationary phase promastigotes were washed three times in HBSS, suspended in complete RPMI, and incubated with the phagocytes. Control phagocytes were cultured in medium alone.

### Thioglycollate-induced peritonitis and leukocyte exit from the peritoneal cavity to the draining lymph node

Peritonitis was induced in BALB/c, C57BL/6 and C57BL/6 SJL mice by injecting 3 ml of a 3% (wt/vol) thioglycollate (Sigma-Aldrich Brasil Ltda, São Paulo Brazil) sterile solution. Three to four days after peritonitis induction, the PEC were collected by a standardized washing of the peritoneal cavity, performed twice with cold Ca^2+^- and Mg^2+^-free Hanks’ balanced salt solution (HBSS, Sigma) containing 20 IU/ml heparin. The number of cells collected from each animal was estimated using a Neubauer chamber. Cell viability was assessed using the trypan blue exclusion technique, and cell populations were defined morphologically using cytospin preparations and by phenotyping using specific antibodies in flow cytometry. Parathymic lymph nodes were collected by dissection and were studied by flow cytometry.

### Rate of *Leishmania*phagocytosis by PEC

To estimate the *Leishmania* phagocytosis rate by different phagocyte populations, parasites were labeled with PKH26 before coincubation with PEC. Briefly, 10^8^ parasites were suspended in 2 ml of PBS, mixed with a 4 μM PKH26 solution in 2 ml of PBS and incubated for 5 min at room temperature in the dark under periodic agitation. Staining was terminated by the addition of 4 ml of fetal bovine serum (FBS), incubation at 37°C for 30 min and washing three times in HBSS. At the end of 24 h of co-culture, the cells were washed and subjected to flow cytometry for identification of the mononuclear phagocyte populations positive for PKH26.

### Adoptive cell transfer-and-tracking experiments

Thioglycollate peritonitis was induced in C57BL/6 (H-2^b^) mice. Three days after peritonitis induction, PEC were collected, washed twice in HBSS, suspended at 2 × 10^6^ cell/mL in RPMI (Sigma) with 10% fetal bovine serum (FBS; Cultilab, Brazil), 60 g/ml gentamicin and 2 mM glutamine (complete RPMI) and cultured alone or with 2 × 10^7^ stationary phase *L. amazonensis* promastigotes for 18–24 h in non-adherent polypropylene tubes at 37°C and 5% CO_2_. The cells were washed and intraperitoneally transferred (1 × 10^7^ cells) to BALB/c (H-2^d^) mice. On the following day, the recipient animals were killed, and the PEC and parathymic lymph nodes were removed. The proportion of H-2^b+^ (C57BL/6) phagocytes of different populations present in the peritoneal cavity and in the draining lymph node was studied by flow cytometry.

To avoid the potential effect of the MHC mismatch between the mice strains, though the migration experiment was performed for only a short period, a similar set of experiments was performed using cell transfer-and-tracking experiments using C57BL/6 Ly5.1^+^ mice cell transfer to C57BL/6 Ly5.1^-^ mice. Three days after peritonitis induction, PEC were collected and intraperitoneally transferred (1 × 10^7^ cells) from C57BL/6 Ly5.1^+^ to C57BL/6 Ly5.1^-^ recipient animals. After 24 h, the PEC and parathymic lymph nodes were removed and processed for flow cytometric analysis.

### Cell preparation and flow cytometric analysis

PEC were washed, counted and resuspended in FACS buffer containing an anti-Fc monoclonal antibody for 30 min on ice to block non-specific staining. Parathymic lymph nodes were cut into small fragments, digested with collagenase D (0.5 mg/ml, Boehringer-Mannheim, Germany) and diluted in RPMI 1640 medium supplemented with 3% FBS and 0.1% DNase I (Roche Diagnostic, Germany) for 10 minutes at 37°C with continuous agitation. Cell suspensions were filtered through gauze and washed twice in a phosphate-buffered saline (PBS) solution supplemented with 5% FBS and 5 mM EDTA containing 5 mg/ml DNase I [7]. The cells were washed, counted and resuspended in FACS buffer for 30 min on ice to block non-specific staining.

The cells (1–2 × 10^6^/stain) were incubated with fluorescein-conjugated antibodies (anti-CD11b (M1/70) and anti-I-A^d^ (AMS-32.1) (BD-Pharmingen; San Jose, CA, USA); anti-F4/80 antigen (R1-2; Caltag; San Diego, CA, USA)), phycoerythrin (PE) conjugates (anti-F4/80 (R1-2; Caltag, USA) and anti-H2^b^ (AF6-88.5, BD-Pharmingen; San Jose, CA, USA), and biotin-conjugated antibodies (anti-CD11c (HL3), CD45.1 (A20; BD-Pharmingen; San Jose, CA, USA) followed by streptavidin Cy-5 or phycoerythrin conjugate (BD-Pharmingen; San Jose, CA, USA). All antibody incubations were performed at 4°C for 20 min and were followed by three washes with FACS buffer. Unlabeled or isotype-matched stained cells were used as a control. Cells were analyzed on a FACScan flow cytometer using CellQuest software (Becton-Dickinson). The histograms and bitmaps of the distributions of cell populations were constructed using FlowJo Software (Tree Star, Inc.). Fifty thousand events were analyzed per sample [12]. The parameters for defining the cell populations have been described in previous studies [21, 25–28]. In brief, cells positive for CD11b and antigens F4/80 were defined as macrophages, populations of cells with high granularity expressing GR-1 were defined as neutrophils, expression of CD11c^hi^ CD11b^hi^ were defined as myeloid dendritic cells.

### Expression and analysis of the results

The numerical data shown in the text, tables and graphs represent percentages of the total number of cells identified according to the defined parameters. To adjust the data obtained in different experiments, the proportion of migrating cells was expressed as the percentage of cells of a given population among the migrating leukocytes (injected into the peritoneal cavity and tracked to the lymph node) relative to the percentage of the same injected cell population remaining in the peritoneal cavity. The significance of the differences observed between the control and infected groups was tested using the paired Student’s t-test. The threshold for statistical significance was set at P < 0.05. All experiments were performed independently a minimum of three times, using a minimum of six animals in each experimental group.

## Results

### *Leishmania*phagocytosis by different phagocyte populations present in the PEC

On the 4^th^ day after thioglycollate injection, the number of cells present in the peritoneal cavity was 1.7 × 10^7^, approximately five times higher than that observed in control animals injected with saline (0.3 × 10^7^ paired t test, P < 0.0001; data not shown). To investigate the efficiency of *Leishmania* phagocytosis by the different mononuclear phagocytes present in PEC, we co-cultivated PEC with PKH26-labeled promastigotes for 24 h and examined the phenotypes of PKH26-labeled leukocytes by flow cytometry. The phagocytosis rate was high (68 ± 31% to 77 ± 22%) among the CD11b^+^ populations (Table 1). However, this rate was substantially low in non-myeloid populations, including non-myeloid DC populations (CD11c^+^ CD11b^-^, 33 ± 13%, Table 1).

**Table 1:** **Infection rate of different population of inflammatory mononuclear phagocytes coincubated** ***in vitro*** **with** ***L. amazonensis***

	Infection ratio (%)
CD11c^+^	72 ± 17
CD11c^+^ CD11b^+^	77 ± 22
CD11c^+^ CD11b^-^	33 ± 13
CD11c^-^ CD11b^+^	68 ± 31
CD11c^-^ CD11b^-^	30 ± 19

Data are the mean (SEM) from seven experiments.

### Effect of *L. amazonensis*infection on the migration of different phagocyte populations from the inflammatory site to the draining lymph nodes

**Figure 1:**
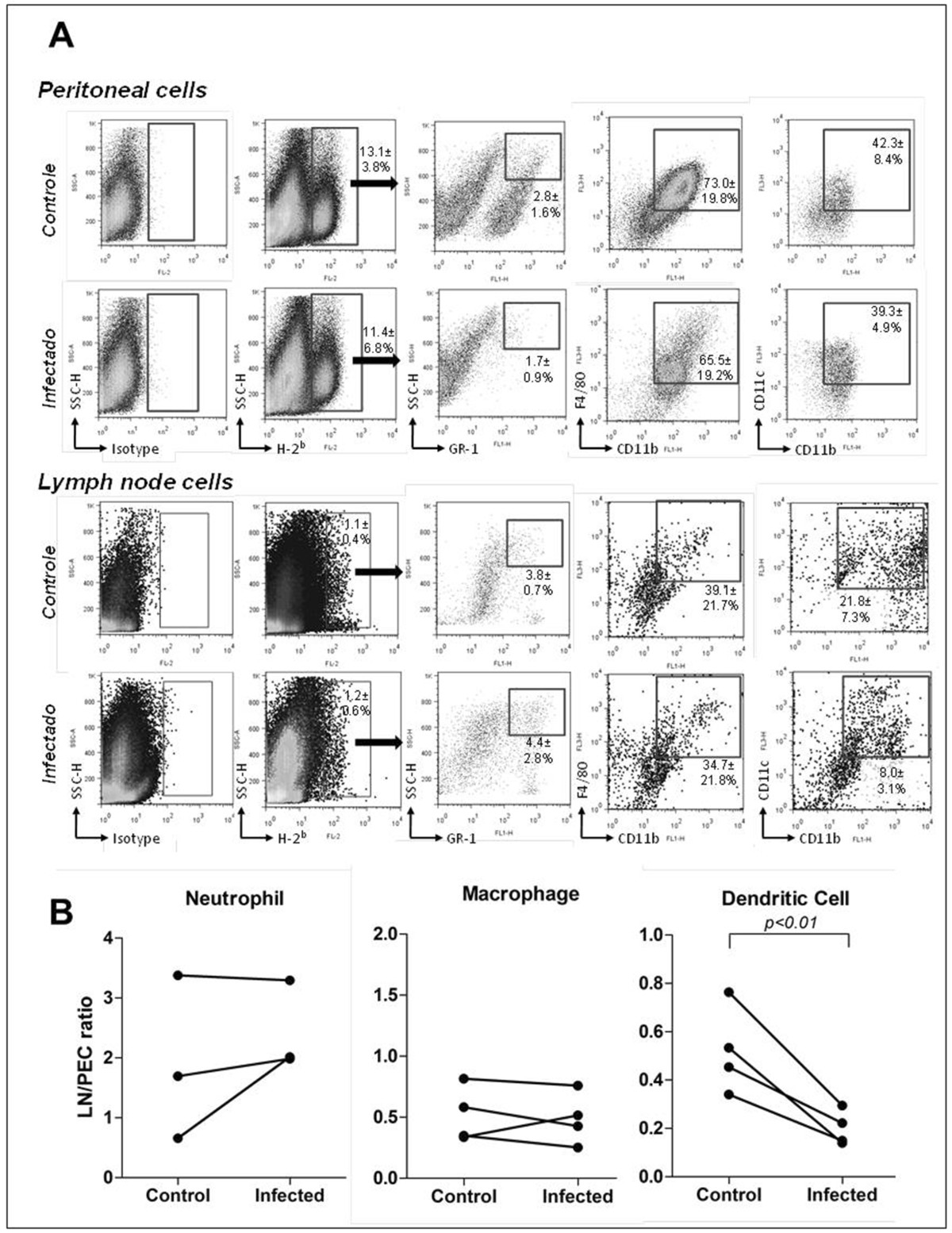
**Phenotypic analysis of the** ***in vivo*** **cell transfer-and-tracking assay from C57BL/6 mice to BALB/c mice.** Infected (*L. amazonensis*) or control (medium alone) thioglycollate-elicited peritoneal exudate cells (PEC) of C57BL/6 mice were injected *i.p.* in BALB/c mice previously stimulated with thioglycollate. After 24 hours, the PEC and draining thymic LNs were collected, and the H-2Kb^+^ (C57BL/6) cells were analyzed. The main phagocyte populations were defined by the expression of the indicated markers. **(A)** Dot plots from one representative experiment with values representing the mean ± sd of four experiments that were independently performed. **(B)** Comparison of the proportion of migrating phagocytes of different phenotypes from the inflammatory site to the draining LN after incubation with medium alone (control) or with *L. amazonensis* (infected) (10 promastigotes per PEC). Each point represents the percentage of cells among the migrating leukocytes (injected into the peritoneal cavity and tracked to the LN) relative to the percentage of the same injected cell population remaining in the peritoneal cavity.

C57BL/6 (H-2^b^) mouse peritoneal donor cells were cultivated overnight with medium alone (control) or with *L. amazonensis* (infected) and then injected into the peritoneal cavity of BALB/c mice (see Methods). After 24 h, the recipient mice injected with donor leukocytes cultivated with medium alone (control) had 13.1 ± 3.8% of H2^b+^ (C57BL/6) cells in the peritoneal cavity. Among these cells, 2.8 ± 1.6% were neutrophils (SSC^hi^ GR-1^+^), 73.0±19.8% were macrophages (CD11b^+^ F4/80^+^) and 42.3±8.4% were myeloid DCs (CD11c^+^ CD11b^+^). In the lymph nodes (LNs) of these animals, 1.1 ± 0.4% of the cells were H2^b+^. Among these migrating cells, 3.8 ± 0.7% were neutrophils (SSC^hi^ GR-1^+^), 39.1±21.7% were macrophages (CD11b^+^ F4/80^+^) and 21.8±7.3% were myeloid DCs (CD11c^+^ CD11b^+^) (Figure 1A).

In the group of animals injected with donor PEC cultivated with *L. amazonensis* (infected), the proportion of the H2^b+^ cells in the peritoneum was 11.4 ± 6.8%. Among these cells, 1.7 ± 0.9% were neutrophils (SSC^hi^ GR-1^+^), 65.5±19.2% were macrophages (CD11b^+^ F4/80^+^) and 39.3±4.9% were myeloid DC (CD11c^+^ CD11b^+^). In the LNs, the proportion of migrating (H2^b+^) cells was 1.2 ± 0.6%. Among these cells, 4.4±2.8% were neutrophils (SSC^hi^ GR-1^+^), 34.7±21.8% were macrophages (CD11b^+^ F4/80^+^) and 8.0±3.1% were myeloid DCs (CD11c^+^ CD11b^+^) (Figure 1A). Only the fraction of migrating myeloid DCs was consistently decreased in the draining LN after co-incubation with *Leishmania* (t Test paired test, P<0.01, Figure 1B).

**Figure 2:**
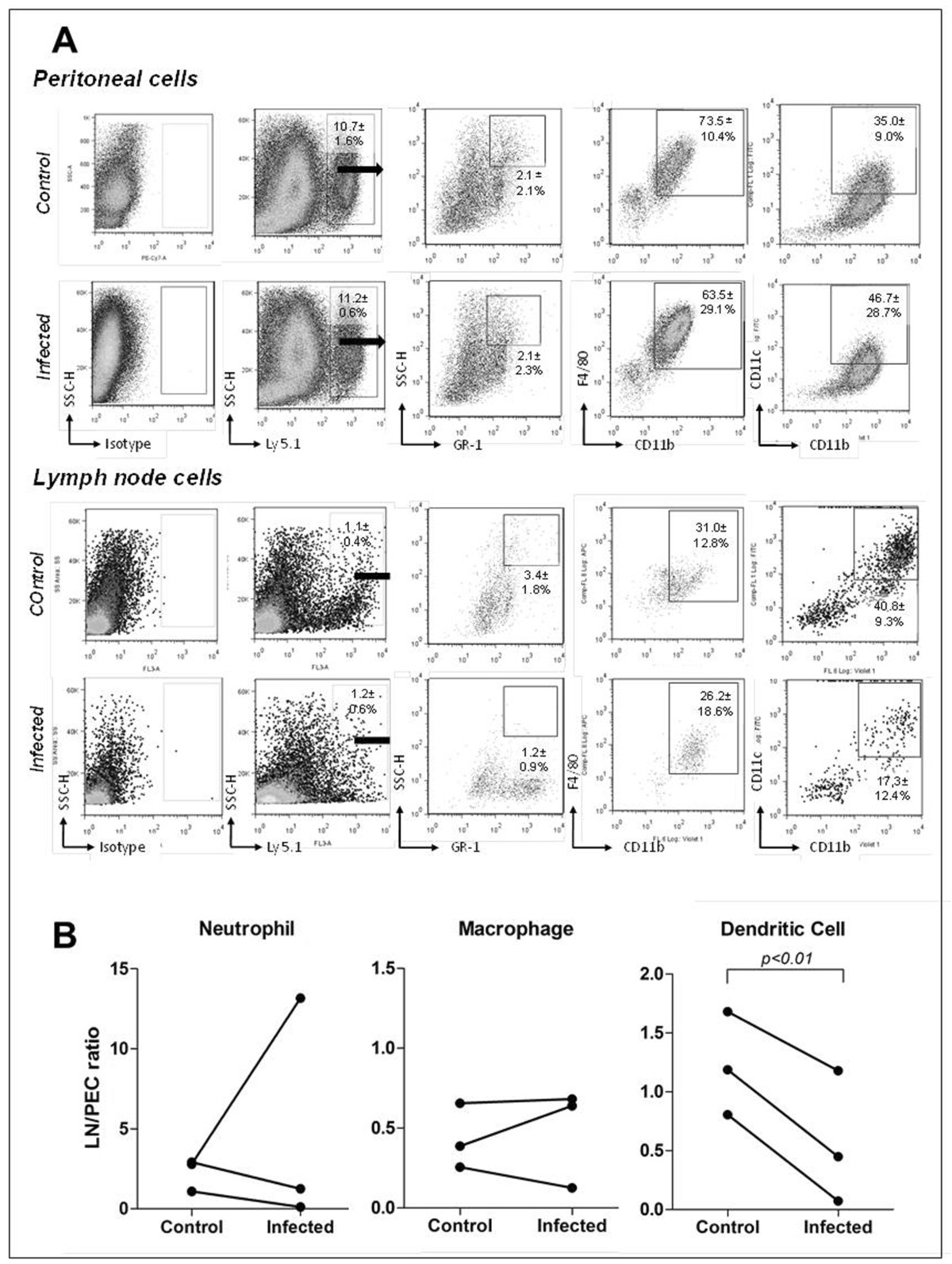
**Phenotypic analysis of Ly5.1** ^**+**^ **cells that migrated from the peritoneum to the LN after** ***Leishmania*** **infection.** Infected (*L. amazonensis*) or control (medium alone) cells from C57BL/6 Ly5.1^+^ mice were injected *i.p.* into C57BL/6 Ly5.1^-^ mice previously stimulated with thioglycollate. After 24 hours, the LNs were collected and only Ly5.1^+^ cells were analyzed. The main phagocyte populations are defined by the expression of the indicated markers. **(A)** Dot plots from one representative experiment with values representing the mean ± sd of three experiments that were independently performed. **(B)** Comparison of the proportion of migrating phagocytes of different phenotypes from the inflammatory site to the draining LN after incubation with medium alone (control) or with *L. amazonensis* (infected) (10 promastigotes per PEC). Each point represents the percentage of cells among the migrating leukocytes (injected into the peritoneal cavity and tracked to the LN) relative to the percentage of the same injected cell population remaining in the peritoneal cavity.

To confirm these data and avoid the potential effects of the MHC mismatch between mice strains, we used a Ly5.1^+^ cell transfer to Ly5.1^-^ animals in the series of experiments shown in Figure 2. C57BL/6 SJL mouse Ly 5.1^+^ peritoneal donor cells were cultivated overnight with medium alone (control) or with *L. amazonensis* (infected) and were then injected into the peritoneal cavity of C57BL/6 Ly 5.1^-^ mice (see Methods). After 24 h, the recipient mice injected with donor leukocytes cultivated with medium alone (control) presented 10.7 ± 1.6% of Ly 5.1^+^ cells in the peritoneal cavity. Among these cells, 2.1 ± 2.1% were neutrophils (SSC^hi^ GR-1^+^), 73.5±10.4% were macrophages (CD11b^+^ F4/80^+^) and 35.0±9.0% were myeloid DCs (CD11c^+^ CD11b^+^). In the LNs of these animals, 1.1 ± 0.4% of the cells were Ly 5.1^+^. Among these migrating cells, 3.4 ± 1.8% were neutrophils (SSC^hi^ GR-1^+^), 31.0±12.8% were macrophages (CD11b^+^ F4/80^+^) and 40.8±9.3% were myeloid DCs (CD11c^+^ CD11b^+^) (Figure 2A).

In the group of animals injected with donor PEC cultivated with *L. amazonensis* (infected), the proportion of the Ly 5.1^+^ cells in the peritoneum was 11.2 ± 0.6%. Among these cells, 2.1 ± 2.3% were neutrophils (SSC^hi^ GR-1^+^), 63.5±29.1% were macrophages (CD11b^+^ F4/80^+^) and 46.7±28.7% were myeloid DCs (CD11c^+^ CD11b^+^). In the LNs, the proportion of migrating (Ly 5.1+) cells was 1.2 ± 0.6%. Among these cells, 1.2 ± 0.9% were neutrophils (SSC^hi^ GR-1^+^), 26.2±18.6% were macrophages (CD11b^+^ F4/80^+^) and 17.3±12.4% were myeloid DCs (CD11c^+^ CD11b^+^) (Figure 2A). Again, only the proportion of migrating (Ly5.1^+^) myeloid DC populations was consistently decreased in the draining LN after co-incubation with *Leishmania* t Test, paired test, P<0.01, Figure 2B.

## Discussion

Our data showed a varied population of leukocytes in the peritoneum at 4 days after thioglycollate-induced peritonitis, with a predominance of cells with a monocyte/macrophage morphology. These data confirm previous observations by Cook and colleagues [29] and were further supported by flow cytometry observations that most of these peritoneal cells had a CD11b^hi^ F4/80^hi^ (macrophage) phenotype. The infection rate and phagocytosis of dead *Leishmania* were high among CD11b^+^ myeloid cells and were similar between CD11b^+^ CD11c^-^ (macrophage/neutrophil) and CD11b^+^ CD11c^+^ (myeloid DC) populations. In contrast, *Leishmania* infection/phagocytosis was low among CD11b^-^ cells, irrespective of CD11c expression. Among the CD11b^-^ CD11c^-^ cells, only 30 ± 19% phagocytized the parasite, and this total may include resident peritoneal macrophages [29]. These data confirm the observations of Murielle and collaborators, who showed that CD11b^+^ and GR-1^+^ cells were the most frequently infected cells present in primary lesions produced by *Leishmania major* infection of the mice hypoderm [4]. Leon and collaborators also showed that both the macrophage (CD11b^+^ CD11c^-^) and the dermal monocyte-derived dendritic cells (CD11b^+^ CD11c^+^) had the highest parasite burdens among the leukocytes found in the dermal-infiltrate after *Leishmania* infection *in vivo* [7]. In a previous study, we also showed that PEC with CD11b^hi^ expression presented the highest levels of *Leishmania* infection compared with the CD11b^neg^ and CD11b^lo^ populations [12]. CD11b is the αM subunit of the integrin capable of mediating interactions of leukocytes with ICAM-1, the extracellular matrix proteins and the complement component iC3b. Therefore, CD11b expression by this myeloid cell population may enhance phagocytosis of opsonized parasites.

In a previous study, we observed that *in vitro* co-incubation for 6–24 h with *Leishmania* decreases the adherence of the J774 cell line, human peripheral blood monocytes and thioglycollate-elicited inflammatory peritoneal exudate cells (PEC) to both connective tissue and connective matrix components, such as fibronectin. This decrease in phagocyte adhesion resulted from an impairment in integrin regulation rather than an inhibition of integrin expression. This decrease also correlated with the percentage of phagocytes containing amastigotes and with the parasite burden in the phagocytes to the extent that, with a 50% phagocyte infection rate and a parasite burden of 4 amastigotes per phagocyte, a 70% reduction in PEC adhesion to fibronectin was achieved [8, 12]. In this study, we show that the coincubation of PEC with *Leishmania* in conditions that favor a high parasite burden also decrease phagocyte migration from an inflammatory site to the draining lymph node *in vivo*. Although a trend of a decrease in the migration rate of F4/80^+^ cells and of neutrophils was observed in some experiments, only the CD11c^+^ cell population presented a consistent decrease in migration in all experiments using the adoptive transfer of cells between C57BL/6 and BALB/c mice or between Ly5.1^+^ and Ly5.1^-^ animals.

Different dendritic cell subsets have been considered the leading transporters of antigens and pathogens from the periphery to the lymph nodes. For instance, Steigerwald and Moll showed that infecting DCs with *L. major* impaired their ability to migrate in response to a variety of chemokines, except to CCL21 [20]. This chemokine is produced by lymphatic endothelial cells and may be involved in leukocyte migration from the inflammatory site to lymph nodes. For instance, monocyte-derived DCs retained in the skin expressed less CCR7 (CCL21 receptor) than did uninfected monocyte-derived DCs that were capable of migrating to the draining lymph nodes [7]. However, Ato and collaborators found that DC migration into the lymphoid tissue of the spleen in *L. donovani*-infected mice was impaired due to deficient CCR7 expression [30]. A possible explanation for these contradictory data is the differences in DC response to infection by different *Leishmania* species. Alternatively, DC populations may present intrinsically different responses to *Leishmania* infection. In our previous work, we have shown that the decrease in phagocyte adherence to connective tissue was independent on the species of *Leishmania*. Furthermore, the parasite burden was directly associated with the decrease in phagocyte adherence to the connective tissue [12]. In this study, we used 10 promastigotes per PEC. This parasite-leukocyte ratio allowed for a high parasite burden and a low level of phagocyte binding to the connective tissue or to isolated connective matrix components in adhesion assays [12]. Therefore, a high parasite burden may impair the migratory DC capability, as occurs with other phagocyte functions, in *Leishmania*-infected cells [12, 17, 20]. Such differences in migration patterns among DC subpopulations and between DC and other phagocyte populations after contact with *Leishmania* deserve further comparative studies using sorted cell populations.

Finally, this finding of the decrease in migration of DCs coincubated with *Leishmania* concurs with studies by Jebbari and colleagues and Leon and colleagues that showed the permanence of infected dendritic cells at the inoculation site [7, 31]. The permanence of these infected cells at the infection site may be a determinant for the appearance of ulcers in tegumentary forms of leishmaniasis. The mechanisms associated with the development of skin lesions in cutaneous localized leishmaniasis are not completely understood. Evidence suggests that these ulcers develop at the site of parasite inoculation. Type IV cellular hypersensitivity may be involved, but in a considerable number of patients, it is not detected by the leishmanin skin test at the time of ulcer [32]. Recently, a subpopulation of DCs that has been implicated in maintaining the inflammatory status in tissues was evidenced in different diseases [33]. Hence, the study of the mechanisms controlling the migration of infected cells and the potential role of migrating and non-migrating DC in the development of lesions is important to understand the pathogenesis of inflammatory diseases.

## Conclusions

Coincubation with *Leishmania* impair DC migration from the inflammatory site to the draining lymph node. Such changes in dendritic cell migration pattern may be associated with immunological events that maintain inflammation at the sites of infection.

## Authors’ original submitted files for images

Below are the links to the authors’ original submitted files for images. Authors’ original file for figure 1 Authors’ original file for figure 2

## Abbreviations

DC: Dendritic cell
PEC: Peritoneal exudate cells
VLA-4: Very Late Antigen-4
HBSS: Hanks’ balanced salt solution
FBS: fetal bovine serum
RPMI: Roswell Park Memorial Institute
LNs: Lymph nodes.
