# Supplementary figures and images for: Leishmania amazonensis infection impairs dendritic cell migration from the inflammatory site to the draining lymph node
Source: BMC Infect Dis. 2014 Aug 20;14:450. doi: 10.1186/1471-2334-14-450 (PMC4143564; doi:10.1186/1471-2334-14-450)

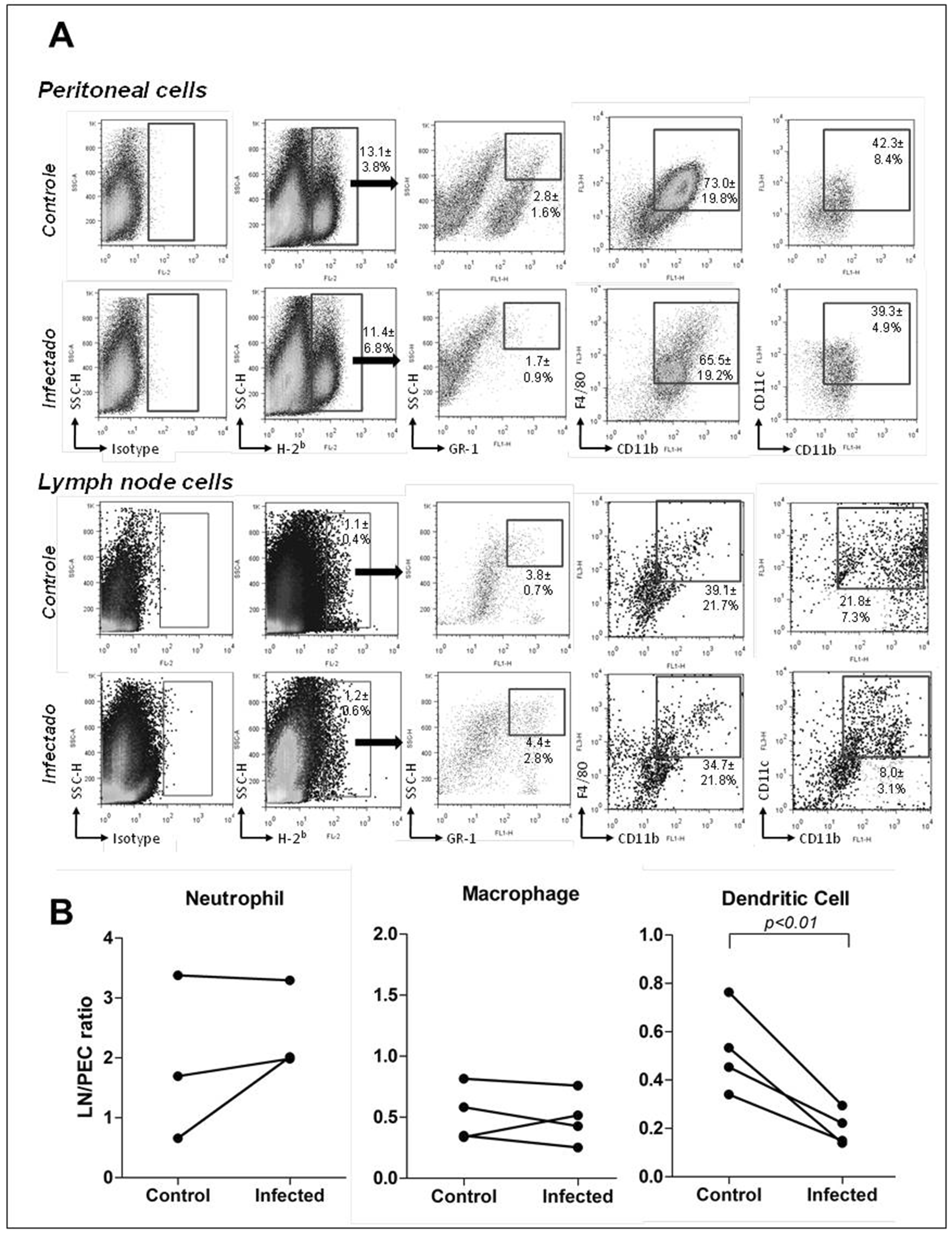

Supplement: Supplementary file 1 — Authors’ original file for figure 1 [file 12879_2014_3744_MOESM1_ESM.tif]

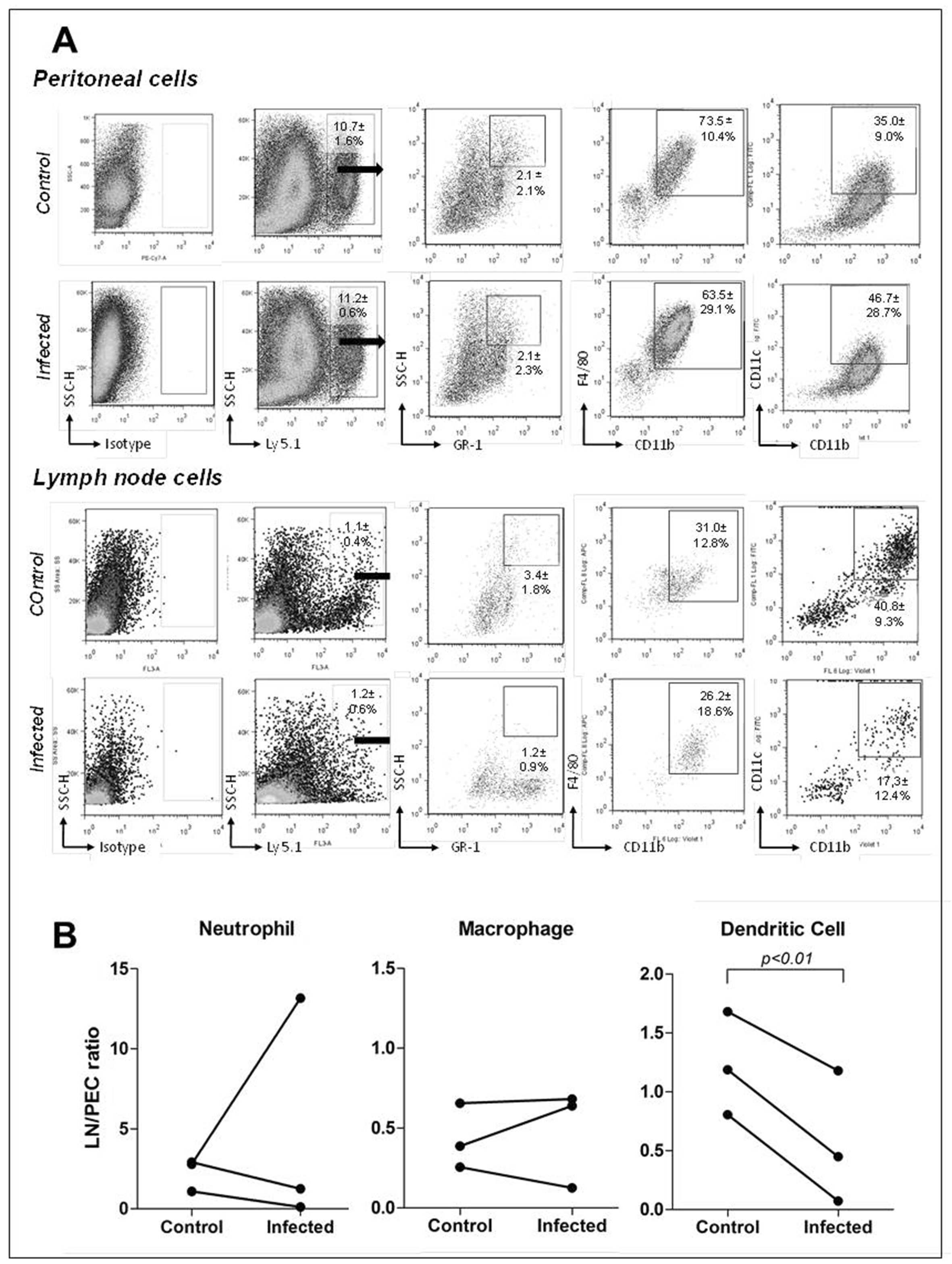

Supplement: Supplementary file 2 — Authors’ original file for figure 2 [file 12879_2014_3744_MOESM2_ESM.tif]
